# Risk, Predictive Factors, and Nomogram of Liver Metastatic Gastroesophageal Junction Cancer: A New Study Based on the Surveillance, Epidemiology, and End Results Database

**DOI:** 10.7759/cureus.63429

**Published:** 2024-06-28

**Authors:** Chenrui Tian, Yang Li, Min Li

**Affiliations:** 1 Oncology, Xinxiang Medical University, Xinxiang, CHN; 2 Pathogenic Biology, School of Basic Medical Sciences, Xinxiang Medical University, Xinxiang, CHN

**Keywords:** nomogram, seer database, overall survival, liver metastasis, cardia

## Abstract

Background and objectives: Liver metastases are associated with a poor prognosis in gastroesophageal junction (GEJ) cancer patients. The high rate of liver involvement is attributed to the unique anatomical location of the GEJ, which is close to the liver. Patients with liver metastasis typically have advanced, unresectable disease and limited treatment options. Therefore, early detection and prediction are crucial to guide appropriate treatment planning and improve the outcomes for patients with GEJ cancer at risk of liver metastases. Using data from the Surveillance, Epidemiology, and End Results (SEER) database, the present study aimed to elucidate the incidence and risk factors of liver metastases in GEJ cancer patients diagnosed between 2010 and 2019.

Methods: This research employed univariable and multivariable logistic regression models to identify risk factors for the development of liver metastases. A predictive nomogram for liver metastases was developed and assessed. Patients' overall survival (OS) with liver metastases was analyzed using the Kaplan-Meier method.

Results: The study included 1,322 eligible patients with GEJ cancer, 181 (13.6%) of whom were diagnosed with liver metastases. The median overall survival (mOS) for patients with liver metastasis was approximately eight months, compared to a shorter mOS for patients without liver metastasis (P < 0.001). Factors significantly associated with the occurrence of liver metastasis included N3 stage (OR: 1.84; 95% CI: (1.13-2.96); P < 0.001), surgery (OR: 0.09; 95% CI: (0.06-0.14); P < 0.001), lung metastasis (OR: 2.88; 95% CI: (1.78-4.63); P < 0.001), chemotherapy (OR: 0.54; 95% CI: (0.32-0.87); P < 0.001), and radiation therapy (OR: 0.33; 95% CI: (0.25-0.45); P < 0.001). The nomogram demonstrated good performance in predicting liver metastases in GEJ cancer patients (c-index: 0.820).

Conclusions: The study identified lymph node status, surgical, lung metastasis, chemotherapy, and radiation as important predictors of outcomes for patients with GEJ cancer. The developed nomogram might be a valuable tool for predicting the risk of liver metastases in GEJ cancer patients, potentially enhancing clinical decision-making processes. By predicting the risk of liver metastasis occurrence, clinicians might intervene in patients with GEJ cancers as early as possible.

## Introduction

Gastroesophageal junction (GEJ) cancer is a type of stomach cancer located near the junction of the esophagus and stomach. It was previously referred to as cardia cancer. The cardia is a physiological sphincter muscle that controls the passage of food into the stomach, while the GEJ is a transitional area without a distinct sphincter function. In recent decades, an increasing trend has been observed in the prevalence of adenocarcinoma of the GEJ, which has greater metastatic probability and worse survival than most cancers [1,2]. In the Western world, patients with GEJ cancer are rare, and the majority are diagnosed at an advanced stage with poor prognosis [3]. Data from the SEER database of the National Cancer Institute (NCI) indicated that the incidence of GEJ cancer increased 2.5-fold over the past 35 years, and now it has become a public health problem. The prognosis of patients with metastatic disease is dismal [4]. Some studies showed that five-year survival rates could be improved without distant metastatic disease [5,6]. However, liver metastasis continues to be among the most frequent sites of distant spread in GEJ cancer [7], so early diagnosis and optimal intervention in patients with liver metastasis are urgently needed. The nomogram might be an effective screening tool for predicting GEJ cancers with liver metastasis. GEJ cancers with liver metastasis have a poor prognosis. We added treatment modalities to the predictive model for the risk of liver metastasis occurrence. By predicting and screening the risk of liver metastasis occurrence, clinicians might intervene in patients with GEJ cancers as early as possible. This article was previously posted to the Research Square preprint server on December 12, 2023.

## Materials and methods

**Figure 1:**
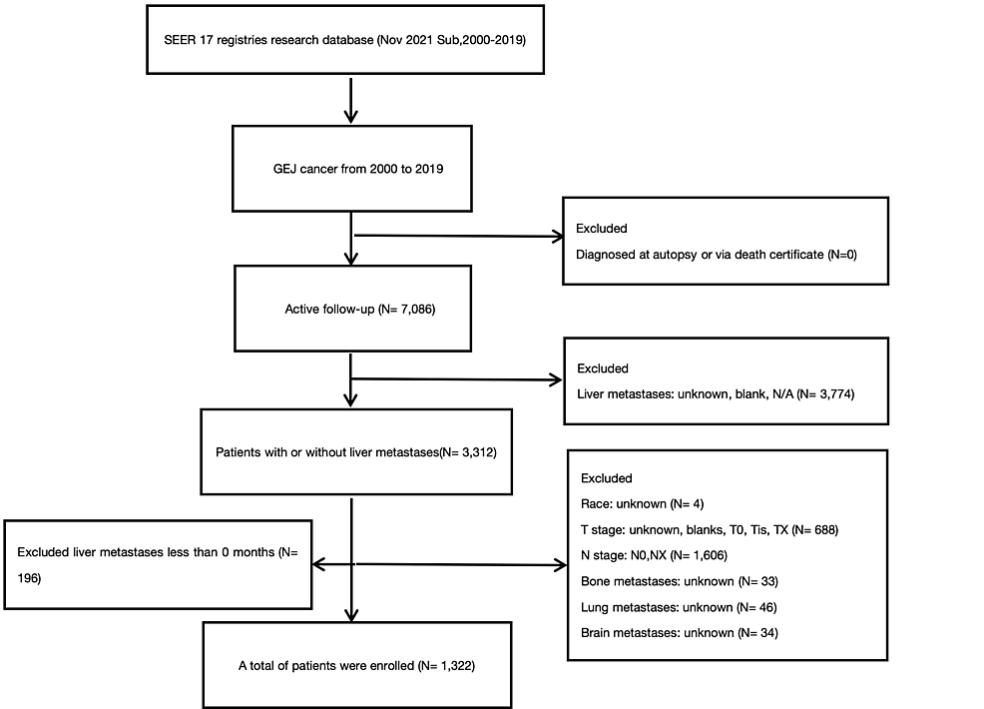
The specific flowchart for the study population selection

Patients diagnosed with GEJ cancer were identified from the SEER database, spanning the years 2010 to 2019, across 17 registries. Inclusion criteria included a primary site labeled "EsophagusGEJunction" and staging information per the American Joint Committee on Cancer (AJCC) seventh edition, tumor, node, metastasis (TNM) classification. Patients with incomplete data, those not classified as N0 stage, and those with missing follow-up information were excluded. Data for each patient included demographic variables (age, sex, and race), clinical variables (tumor grade, AJCC 7th edition TNM staging, and sites of metastasis), treatment variables (chemotherapy, radiation therapy, surgical procedures on the primary site, and scope of regional lymph node surgery), and outcome variables (duration of survival and vital status). Variables with unknown or unclear classifications were excluded from the analysis. The selection process is detailed in Figure 1.

Statistical analyses were performed using R software (version 4.3.1). A P-value of <0.05 (two-sided) was deemed statistically significant. Demographic and clinical data collected encompassed age; sex; race; grade; TNM stages; presence of lung, brain, and bone metastases; surgery; chemotherapy; radiation; regional lymph node dissection, and survival duration. Pearson’s chi-square or Fisher’s exact tests were employed to analyze differences in liver metastasis incidence among categorical variables. Forward and backward selection methods determined relevant variables for the "rms" package to develop a GEJ cancer nomogram for liver metastasis risk prediction. Calibration curves evaluated the nomogram's accuracy, while Harrell's C-index assessed its discriminatory ability. Bootstrapping validation (1000 resamples) refined the c-index. OS was analyzed using the Kaplan-Meier method and the log-rank test.

## Results

Baseline characteristics of the study population

A total of 7,086 GEJ cancer patients were initially identified. Of that, 1,089 were male and 233 were female. The majority of patients were aged above 60 years. Among all patients, 181 were diagnosed with liver metastasis. The predominant race in the non-liver metastasis group was Caucasian (N = 977; 85.6%). Most patients were diagnosed at stages T3-T4 (N = 857; 75.1%) and N1 (N = 766; 67.1%). Of the 181 patients with liver metastasis, 20 (11.0%) had bone metastasis, 28 (15.5%) had lung metastasis, and four (2.2%) had brain metastasis. Overall, 694 (52.5%) patients underwent surgery, and 1,134 (85.8%) received chemotherapy. Finally, 1,322 patients without missing data were included in the univariable and multivariable logistic regression analyses, and a nomogram predicting liver metastasis was developed. The chi-square test confirmed the complete randomization of deviations. The clinical and demographic characteristics of these 1,322 patients are detailed in Table 1.

**Table 1:** Clinical characteristics of 1,322 GEJ cancer patients diagnosed from 2010 to 2019 LiverM: Liver metastases; RLN: Regional lymph node dissection. A p-value less than 0.05 is considered to be statistically significant. P < 0.05*; P < 0.01**; P < 0.001***.

	Without LiverM (N = 1141)	With LiverM (N = 181)	x^2^	P-value
Age (years)			2.75	0.25
<50	120 (10.5%)	21 (11.6%)		
50-60	246 (21.6%)	48 (26.5%)		
≥60	775 (67.9%)	112 (61.9%)		
Sex			0.08	0.76
Female	203 (17.8%)	30 (16.6%)		
Male	938 (82.2%)	151 (83.4%)		
Race			NA	0.03
American Indian/Alaska	9 (0.8%)	1 (0.6%)		
Asian or Pacific Islander	110 (9.6%)	11 (6.1%)		
African American	45 (3.9%)	15 (8.3%)		
Caucasian	977 (85.6%)	154 (85.1%)		
Grade			0.25	0.61
G1-2	403 (35.3%)	68 (37.6%)		
G3-4	738 (64.7%)	113 (62.4%)		
T			6.61	0.01**
T1-2	284 (24.9%)	62 (34.3%)		
T3-4	857 (75.1%)	119 (65.7%)		
N			4.35	0.11
N1	766 (67.1%)	131 (72.4%)		
N2	247 (21.6%)	27 (14.9%)		
N3	128 (11.2%)	23 (12.7%)		
Bone metastases			22.09	<0.001***
No	1105 (96.8%)	161 (89.0%)		
Yes	36 (3.2%)	20 (11.0%)		
Lung metastases			47.374	<0.001***
No	1104 (96.8%)	153 (84.5%)		
Yes	37 (3.2%)	28 (15.5%)		
Brain metastases			NA	0.05*
No	1134 (99.4%)	177 (97.8%)		
Yes	7 (0.6%)	4 (2.2%)		
Surgery			138.74	<0.001***
No	468 (41.0%)	160 (88.4%)		
Yes	673 (59.0%)	21 (11.6%)		
Chemotherapy			3.16	0.07
No	154 (13.5%)	34 (18.8%)		
Yes	987 (86.5%)	147 (81.2%)		
Radiation			66.43	<0.001***
None/Unknown	369 (32.3%)	116 (64.1%)		
Yes	772 (67.7%)	65 (35.9%)		
Regional lymph node dissection			NA	<0.001***
None	468 (41.0%)	159 (87.8%)		
1-3 RLN	26 (2.3%)	1 (0.6%)		
4 or more RLN	647 (56.7%)	21 (11.6%)		

Risk factors for developing liver metastasis and survival outcome

The 7,086 GEJ cancer patients diagnosed from 2010 to 2019 were extracted to estimate the risk factors for developing liver metastasis. Univariable logistic analysis showed that the factors of T3-4 stage (OR: 1.25; 95% CI: (0.98-2.27); P = 0.008), N2 stage (vs. N0; OR = 0.63; 95% CI: (0.43-0.91)), surgery (vs. No; OR = 0.09; 95% CI: (0.06-0.13); P < 0.001), radiation (vs. No/unknown; OR = 0.26; 95% CI: (0.20-0.35); P < 0.001), chemotherapy (vs. No/unknown; OR = 0.67; 95% CI: (0.48-0.95); P < 0.001), one to three regional lymph node dissection (vs. No/Unknown; OR = 0.11; 95% CI: (0.01-0.43); P < 0.001), four or more regional lymph node dissection (vs. No/Unknown; OR = 0.09; 95% CI: (0.06-0.13); P < 0.001) were negatively associated with liver metastasis occurrence. However, the male patients (OR: 1.27; 95% CI: (1.13-1.43); P < 0.001), P = 0.04) and N3 stage (vs. N0; OR = 1.05; 95% CI: (0.69-1.55); P < 0.001) were positively associated with liver metastasis risk (Table 2).

**Table 2:** Univariable logistic regression for analyzing the risk factors for developing liver metastases in GEJ cancer patients A p-value less than 0.05 is considered to be statistically significant. P < 0.001*. GEJ: Gastroesophageal junction.

	Univariable OR (95% CI)	z	P-value
*Age (years)*			
<50	Reference		
50-60	1.11 (0.70-1.80)	0.3	0.457
≥60	0.83 (0.54-1.28)	-0.7	0.702
*Sex*			
Female	Reference		
Male	1.27 (1.13-1.43)	-9.7	<0.001*
*Race*			
American Indian/Alaska	Reference		
Asian or Pacific Islander	1.28 (0.76-2.26)	-0.09	0.92
Caucasian	0.85 (0.52-1.46)	0.33	0.74
African American	0.71 (0.36-1.41)	1.00	0.31
*Grade*			
G1-2	Reference		
G3-4	0.15 (0.13-0.18)	0.5	0.57
*T*			
T1-2	Reference		
T3-4	1.25 (0.98-2.27)	2.6	0.008
*N*			
N1	Reference		
N2	0.63 (0.43-0.91)	-2.0	0.04
N3	1.05 (0.69-1.55)	0.2	<0.001*
*Bone metastases*			
No	Reference		
Yes	3.81 (2.33-6.11)	4.5	<0.001*
*Lung metastases*			
No	Reference		
Yes	5.46 (3.51-8.42)	6.4	<0.001*
*Brain metastases*			
No	Reference		
Yes	2.73 (1.91-3.87)	2.0	0.04
*Surgery*			
No	Reference		
Yes	0.09 (0.06-0.13)	-9.9	<0.001*
*Chemotherapy*			
No	Reference		
Yes	0.67 (0.48-0.95)	-1.8	<0.001*
*Radiation*			
No	Reference		
Yes	0.26 (0.20-0.35)	-7.8	<0.001*
*Regional lymph node dissection*			
No	Reference		
1-3	0.11 (0.01-0.43)	-2.1	0.03
4 or more	0.09 (0.06-0.13)	-9.7	<0.001*

The multivariable analysis further confirmed that liver metastasis was positively associated with N3 stage (OR = 1.84; 95% CI: (1.13-2.96); P = 0.03) and lung metastasis (OR = 2.88; 95% CI: (1.78-4.63); P < 0.001), while surgery (vs. No; OR = 0.09; 95% CI: (0.09-0.14); P < 0.001), radiation (vs. No; OR = 0.33; 95% CI: (0.25-0.45); P < 0.001), and chemotherapy (vs. No; OR = 0.54; 95% CI: (0.32-0.87); P < 0.001) were negative with liver metastasis (Table 3).

**Table 3:** Multivariable logistic regression for analyzing the risk factors for developing liver metastases in GEJ cancer patients A p-value less than 0.05 is considered to be statistically significant. P < 0.001*. GEJ: Gastroesophageal junction.

	Multivariable OR (95% CI)	z	P-value
N			
N1	Reference		
N2	1.18 (1.01-1.37)	-0.2	0.77
N3	1.84 (1.13-2.96)	1.9	<0.001*
Lung metastases			
No	Reference		
Yes	2.88 (1.78-4.63)	3.4	<0.001*
Surgery			
No	Reference		
Yes	0.09 (0.06-0.14)	-1.6	<0.001*
Chemotherapy			
No	Reference		
Yes	0.54 (0.32-0.87)	1.0	<0.001*
Radiation			
No	Reference		
Yes	0.33 (0.25-0.45)	-6.2	<0.001*

**Figure 2:**
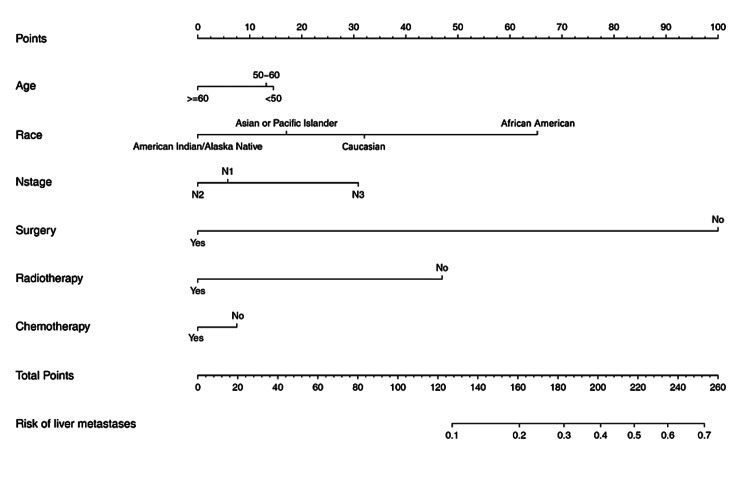
The predicting nomogram for liver metastasis in GEJ cancer patients GEJ: Gastroesophageal junction.

The prediction nomogram that integrated all significant factors for liver metastases in the multivariable logistic regression model was developed (Figure 2).

**Figure 3:**
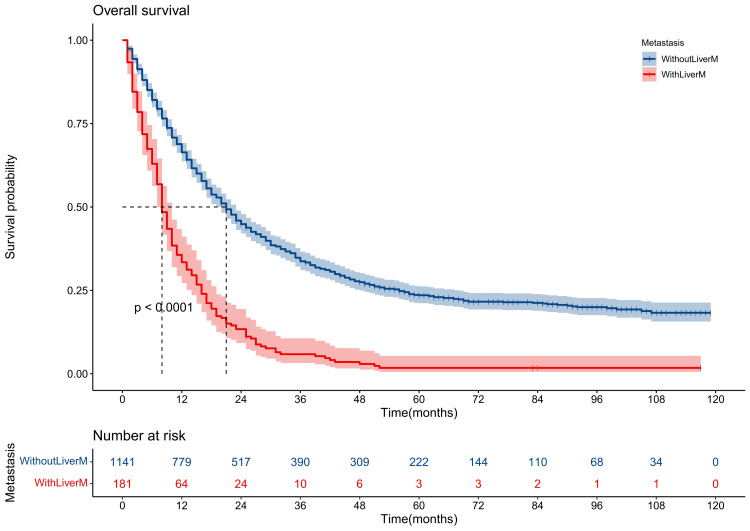
Survival curve for GEJ cancer patients with or without liver metastases A p-value less than 0.05 is considered to be statistically significant. LiverM: Liver metastases; GEJ: Gastroesophageal junction.

The mOS for patients with liver metastasis was about eight months, while the patients without liver metastasis were 20 months (P < 0.001) (Figure 3).

Performance and validation of the nomogram for predicting liver metastasis

**Figure 4:**
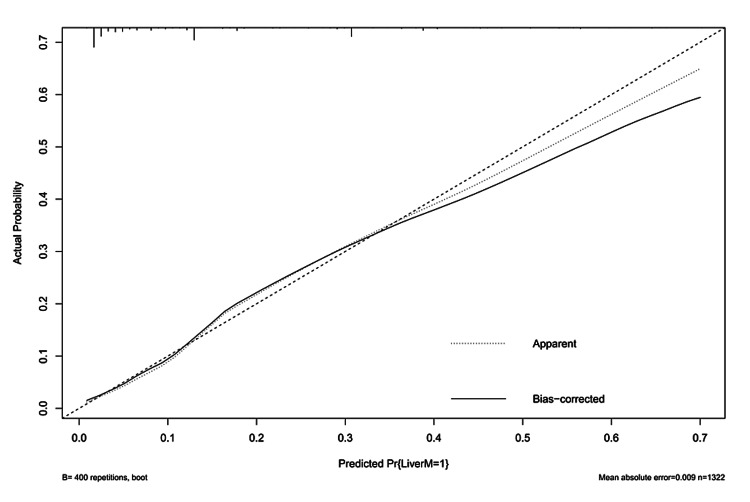
Calibration curve of nomogram LiverM: liver metastases.

To construct the prediction nomogram for liver metastasis, significant factors were identified through a methodical approach using forward and backward stepwise selection. The calibration curve showed good agreement between the predicted and observed probabilities for liver metastasis in GEJ cancer patients (Figure 4).

**Figure 5:**
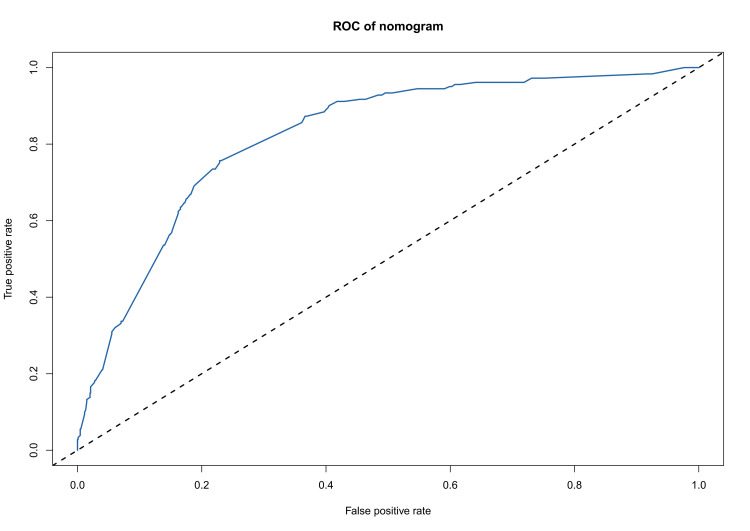
The curve of ROC ROC: Receiver operating characteristic.

The C-index for the prediction nomogram was 0.820. Additionally, receiver operating characteristic (ROC) analysis further substantiated the nomogram's efficacy, with the area under the curve (AUC) also registering at 0.820 (Figure 5).

## Discussion

GEJ cancer is an aggressive tumor located at the junction of the esophagus and stomach. In this study, we used the SEER database to analyze the clinical characteristics and risk factors for liver metastasis in GEJ cancer patients. This is the first time a nomogram has been developed to predict liver metastasis in this patient population. Although this study did not provide a detailed analysis and discussion of higher-grade or lower-grade GEJ cancer, the nomogram would be more suitable as a clinical screening tool. Due to the relatively limited source of the database, all factors classified as unclear or unknown were excluded, resulting in a study cohort of 7,086 GEJ cancer patients, of whom 181 were diagnosed with liver metastasis. Univariable and multivariable logistic regression analyses were conducted to identify the risk factors for liver metastasis development. GEJ cancer occurs more in males than females, following the study of Pakzad et al. and Tan et al. [8,9]. The predominant risk factor for GEJ cancer is gastric reflux leading to Barrett's esophagus, which is more common in males. Although gastroesophageal reflux is equally prevalent in women and men, erosive reflux disease is more frequent in males, with a male-to-female ratio of 1.6. Additionally, chronic bile reflux, smoking, and alcohol intake, which are more common in men, also increase the probability of developing gastroesophageal reflux. While sex differences in tobacco and alcohol consumption are well-established, these behavioral differences alone cannot fully account for the sex disparity in the incidence of GEJ cancer [10].

The differences in race illustrated that Caucasians were significantly more represented than the other races. Buas et al. and Tan et al. also pointed out [4,9] that this outcome was incorrect with our results. Both of them gave several factors such as *Helicobacter pylori* gastroesophageal reflux disease (GERD) and obesity [11]. However, although Caucasians were the majority of 1,322 GEJ cancer patients, they did not have the highest scores. This disparity may result from a combination of factors, including the influence of the environment on gene expression, inequalities in health insurance and access to care, as well as social and economic factors [12].

Treatment modality emerged as a crucial factor in metastasis prevention, with both radiotherapy and chemotherapy serving as effective preoperative or postoperative management strategies for GEJ cancers [13]. The hazard ratio of preventing metastases for patients who received chemotherapy was 0.47 (95% CI: 0.44-0.51) [14]. Therefore, early intervention is very important for preventing liver metastasis of GEJ cancer. ROC analysis showed that the AUCs of the nomogram reached 0.820, which showed good discrimination in predicting the risk of liver patients compared with other risk factors. The study revealed a mOS of eight months for patients with liver metastasis compared to 20 months for those without liver metastasis, corroborating the findings by Chen et al. [15]. Adam et al. found that the mOS was 14 months in patients who had liver metastasis that originated from gastrointestinal primary tumors [16]. This survival advantage varies depending on the primary tumor site, with significant benefits observed in patients undergoing resection for liver metastasis [17]. Additionally, the advantages of our study compared with the existing prognostic nomograms are as follows. First, many studies only focused on esophageal cancer and gastric cancer with and without distant metastasis rather than single metastasis [15,18-20], while our research focused on GEJ cancer with liver metastasis and constructed a predicting nomogram. The predicting value of liver risk reached 0.820 in ROC, which might be very useful for the clinical prediction of liver metastasis and early intervention.

However, the study has several limitations. First, for any cancer, the "primary site" was an important risk factor for intervention and prognosis. The selection variables of "primary site---EsophagusGEJunction", may cause the results incomprehensive. We did not collect other available publicly data on GEJ cancers, which has an inherent bias. Second, we did not perform propensity score matching, which may have led to biased results due to the influence of certain variables. For example, in the distribution of race, Caucasians made up the majority, which could have skewed the predictive results more toward Caucasians. However, since patients with liver metastases were rare, the application of propensity score matching would have resulted in a smaller sample size of included patients, leading to limitations. Third, other factors like tumor markers were not collected in the SEER database, which may reduce the prediction value of the nomogram.

## Conclusions

This study utilized patient data on GEJ cancer from the SEER database between 2010 and 2019 to establish a prognostic nomogram predicting the risk of liver metastasis. Due to the relatively limited source of the database, the results of this study are more inclined toward the patients within this particular database. Furthermore, external data validation is needed to ensure the accuracy of the predictive results. This study might determine that age, grade, N stage, and lung metastasis were the risk factors of liver metastasis for GEJ cancer. The mOS of liver metastasis was eight months. The present SEER study provided insights into the epidemiology of liver metastasis in diagnosed GEJ cancer patients. The nomogram might be used as an intuitive tool to predict and screen liver metastasis in GEJ cancers. Finally, all the conclusions of this study are only applicable to the patients extracted from the 2010-2019 SEER database.
